# Short-Term Associations of Fine Particulate Matter and Synoptic Weather Types with Cardiovascular Mortality: An Ecological Time-Series Study in Shanghai, China

**DOI:** 10.3390/ijerph17031111

**Published:** 2020-02-10

**Authors:** Qing Tian, Mei Li, Scott Montgomery, Bo Fang, Chunfang Wang, Tian Xia, Yang Cao

**Affiliations:** 1 Department of Public Health Sciences, Karolinska Institutet, 17177 Stockholm, Sweden; qing.tian@stud.ki.se; 2 Center for Assessment of Medical Technology, Örebro University Hospital, Örebro University, 70182 Örebro, Sweden; mei.li@regionorebrolan.se; 3 Clinical Epidemiology and Biostatistics, School of Medical Sciences, Örebro University, 70182 Örebro, Sweden; scott.montgomery@oru.se; 4 Clinical Epidemiology Division, Department of Medicine, Karolinska Institutet, 17177 Stockholm, Sweden; 5 Department of Epidemiology and Public Health, University College London, London WC1E 6BT, UK; 6 Division of Vital Statistics, Shanghai Municipal Center for Disease Control and Prevention, Shanghai 200336, China; fangbo@scdc.sh.cn (B.F.); wangchunfang@scdc.sh.cn (C.W.); 7 Institute of Health Information, Shanghai Municipal Center for Disease Control and Prevention, Shanghai 200336, China

**Keywords:** cardiovascular mortality, air pollution, fine particulate matter, PM_2.5_, weather, synoptic weather type, interaction effect, antagonistic effect, synergistic effect, lag effect

## Abstract

*Background*: Exposures to both ambient fine particulate matter (PM_2.5_) and extreme weather conditions have been associated with cardiovascular disease (CVD) deaths in numerous epidemiologic studies. However, evidence on the associations with CVD deaths for interaction effects between PM_2.5_ and weather conditions is still limited. This study aimed to investigate associations of exposures to PM_2.5_ and weather conditions with cardiovascular mortality, and further to investigate the synergistic or antagonistic effects of ambient air pollutants and synoptic weather types (SWTs). *Methods*: Information on daily CVD deaths, air pollution, and meteorological conditions between 1 January 2012 and 31 December 2014 was obtained in Shanghai, China. Generalized additive models were used to assess the associations of daily PM_2.5_ concentrations and meteorological factors with CVD deaths. A 15-day lag analysis was conducted using a polynomial distributed lag model to access the lag patterns for associations with PM_2.5_. *Results*: During the study period, the total number of CVD deaths in Shanghai was 59,486, with a daily mean of 54.3 deaths. The average daily PM_2.5_ concentration was 55.0 µg/m^3^. Each 10 µg/m^3^ increase in PM_2.5_ concentration was associated with a 1.26% (95% confidence interval (CI): 0.40%, 2.12%) increase in CVD mortality. No SWT was statistically significantly associated with CVD deaths. For the interaction between PM_2.5_ and SWT, statistically significant interactions were found between PM_2.5_ and cold weather, with risk for PM_2.5_ in cold dry SWT decreasing by 1.47% (95% CI: 0.54%, 2.39%), and in cold humid SWT the risk decreased by 1.45% (95% CI: 0.52%, 2.36%). In the lag effect analysis, statistically significant positive associations were found for PM_2.5_ in the 1–3 lag days, while no statistically significant effects were found for other lag day periods. *Conclusions*: Exposure to PM_2.5_ was associated with short-term increased risk of cardiovascular deaths with some lag effects, while the cold weather may have an antagonistic effect with PM_2.5_. However, the ecological study design limited the possibility to identify a causal relationship, so prospective studies with individual level data are warranted.

## 1. Introduction

Exposure to air pollution has been widely acknowledged as an important influencing factor for cardiovascular health [1]. In recent years, evolving epidemiological and clinical research has provided convincing evidence that exposure to air pollution, especially to fine particulate matter or PM_2.5_, can lead to progression of cardiovascular disease and triggering of acute cardiac events. PM_2.5_ refers to particulate matters with an aerodynamic equivalent diameter of 2.5 μm or less, which can be suspended in the air and inhaled into the lung to reach the alveoli. The higher its concentration in the air, the more serious is the air pollution. Investigations found that inhalation of air pollutants promotes the development of heart failure, arrhythmia, ischemic heart disease, and high blood pressure, and increases the incidence and mortality of cardiovascular diseases (CVD) [2]. Not only cardiovascular and respiratory diseases, but also all-cause mortality could be partly attributable to exposure to PM_2.5_ air pollution, regardless of the duration of the exposed window period [3,4].

Air pollution is a risk factor that can be modified, and scholars predicted potential mortality benefits of air pollution control in urban China, such that approximately 241,000–1,841,000 life-years could be saved annually for different scenarios of air pollution improvements, indicating substantial health benefits such as a 25% improvement in hypertension control and 30% reduction in cigarette control combined [5].

The effects of air pollution on cardiac function are both long term and short term. A recent international study revealed that an increase of 10 μg/m^3^ in the two-day moving average of PM_2.5_ concentration was associated with increase of 0.55% (95% confidence interval (CI): 0.45%, 0.66%) in daily cardiovascular mortality [6]. A systematic review gathered evidence on the long-term effects of exposure to PM_2.5_, finding that an increase of 10 μg/m^3^ in PM_2.5_ concentration was associated with as high as 11% excess CVD mortality [7]. Furthermore, studies discovered that one of the independent modifiable risk factors triggering cardiovascular deaths was a long-term exposure to particulate matter [8,9]. For short-term effects, a meta-analysis in China summarized the evidence and concluded that 0.68% (95% CI: 0.39%, 0.97%) higher incidence of cardiovascular mortality is related to each 10 µg/m^3^ increase in PM_2.5_ [10]. Evidence from American and European countries also showed similar findings, suggesting that increased PM_2.5_ levels would significantly increase the risk of CVD deaths [4,11].

Meteorological conditions have been shown to influence daily mortality and disease burden in many studies, especially for the cardiovascular system. Weather conditions such as extreme temperature [12], diurnal temperature range [13,14], temperature variation [15], and humidity [16] have been defined as risk factors or effect modifiers that may contribute to CVD mortality.

However, results from research on ambient temperature and cardiovascular events have been inconsistent. A recent review gathered literature on associations of cold and heat on CVD risk, and concluded that both high and low temperatures were associated with higher cardiovascular risk [17]. In contrast, another review suggested that only heat waves might increase cardiovascular mortality, while cold spells increase the morbidity risk [18]. Excessively hot episodes in tropical areas have been shown to increase CVD mortality by 16.63 times (95% CI: 10.47, 26.42) compared with other seasons [19]. A study in Hong Kong identified that cold weather was associated with emergency hospital admission for cardiovascular events, with a relative risk (RR) of 1.22 (95% CI: 1.15, 1.29) [20]. Researchers from Vietnam found a 1 °C decrease in temperature was associated with an increase in CVD admissions of 12% (95% CI: 1%, 25%) [21]. A case series study in Catalonia concluded that cold spells, but not heat waves, increased the incidence of emergency cardiovascular hospitalizations [22].

Other weather factors have also been found associated with cardiovascular health. The presence of extreme weather and sudden decrease in air pressure are associated with more hospital admissions for CVD [23]. Descriptive analysis suggests that both cold, cloudy days and warm, rainy days with high humidity are related to CVD morbidity [24]. Long sunshine duration has been associated with a 15% increased risk of cardiovascular events [25].

Although PM_2.5_ and extreme weather conditions have been widely studied, results from research into CVD mortality associated with synergistic or antagonistic effects between PM_2.5_ and weather conditions remains inconsistent and inconclusive. Many previous studies included meteorological variables as individual covariates, while few of them fully considered the internal relationship of these variables and their collective effects. Importantly, investigations in high-income countries may not be applicable to China, which has one of the largest populations exposed to high levels of air pollution.

Our previous study indicated that PM_2.5_ together with favorable synoptic weather types (SWTs) was significantly associated with higher non-accidental mortality [26]. Based on our previous findings, we wanted to further investigate the associations between CVD deaths and PM_2.5_ pollution and weather conditions using a large-scale database of daily mortality in Shanghai, aiming at providing updated evidence on the associations of CVD mortality with PM_2.5_ and weather conditions, especially the effects of the interaction between air pollutants and the SWT.

## 2. Materials and Methods

### 2.1. Study Design and Setting

The study is observational and ecological, for the time period 1 January 2012 to 31 December 2014, aiming to access the associations of environmental factors with non-accidental CVD deaths. The environmental factors include air pollutants PM_2.5_, nitrogen dioxide (NO_2_), sulfur dioxide (SO_2_), six SWTs, and interactions between PM_2.5_ and SWT.

The population of the study was the residents who were registered in the Household Register of Shanghai (HRS). Shanghai is a municipality and the largest city in the east China, with longitude and latitude of 121° E and 31° N, located in the Yangtze River Delta Region, with a territory of about 6340 km^2^. Shanghai is the most populous city in the east China with a permanent resident population of around 24.18 million in 2017 and a local gross domestic product of approximately 3.27 trillion Yuan in 2017 [27].

### 2.2. Data Collection

Because PM_2.5_ was not routinely monitored in Shanghai until the end of 2012, we obtained hourly PM_2.5_ concentrations in 2012 published by the Shanghai US Consulate General, which was regarded as a reliable source of PM_2.5_ concentrations in China [28]. Daily average PM_2.5_ concentrations from 1 January 2013 to 31 December 2014 were obtained from the Shanghai Meteorological Bureau. The data came from only one air monitor during our study period to present the PM_2.5_ level for the whole city.

Daily weather meteorological data, including temperature, relative humidity, air pressure, wind speed, precipitation, and sunshine hours, during the same period were also obtained from the Shanghai Meteorological Bureau. Weather conditions were categorized into six SWTs using the method introduced by Vanos [29]. The extraction of daily SWT was based on the cluster analysis using measurements of temperature, barometric pressure, humidity, sunshine time, precipitation, and wind velocity, etc. constituting 18 meteorological variables during a day. The final weather types identified in our study included: hot dry, warm humid, cold dry, cool dry, cool humid, and cold humid [26].

Daily CVD mortality data in Shanghai were obtained from the Causes of Death Register of Shanghai (CDRS) provided by the Shanghai Municipal Center for Disease Control and Prevention (SCDC). Mortality from major CVDs was identified according to the International Classification of Diseases, 10th edition (ICD-10) codes I00-I78. There were no district-specific data available in the current study. CVD mortality was represented using daily death counts. Because the total population was relatively stable during the study period, we treated it as if it remained unchanged to produce mortality rates.

### 2.3. Statistical Analyses

Descriptive statistical methods were used to describe the characteristics of the variables. Daily CVD mortality and daily concentrations of air pollutants were examined graphically using time-series plots.

Generalized additive models (GAM) were used to assess the associations of exposure to PM_2.5_ and SWT with CVD mortality [30,31,32]. The regression models included the following components:

(1) The main risk factors PM_2.5_ and (2) SWT, using an indicator variable; (3) an interaction term between them; (4) an indicator variable for day of the week (DOW); and (5) a smooth function *S* for time to control for seasonal trend and unobserved confounders. The final model is described below, which was evaluated using Akaike’s information criterion (AIC):

(1) $$\log\left[E\left(Y_{t}\right)\right]=\beta_{0}+\beta_{1}\cdot PM_{2.5,t}+\beta_{2}\cdot SWT_{t}+\beta_{3}\cdot PM_{2.5,t}\times SWT_{t}+\beta_{4}\cdot DOW_{t}+S\left(t\right),$$

where *E*(*Y_t_*) refers to the expected cardiovascular deaths on day *t*; *β*_0_ represents the intercept; *β*_1_–*β*_4_ refer to the coefficients for each variable; *PM*_2.5,*t*_ represents the daily average PM_2.5_ concentration on day *t*; *DOW_t_* represents a 6 × 1 vector of DOW for day *t*; *SWT_t_* denotes a 5 × 1 vector of SWT for day *t*; PM_2.5,*t*_ × *SWT_t_* denotes the interaction between PM_2.5_ and SWT for day *t*. *S*(*t*) is a smoothing function implemented by cubic B-splines, giving associations with unobserved factors and seasonal trends [33]. According to the AIC, we used a total of 45 knots for the splines to present the time trend and capture the true underlying parameters.

We also examined the 15-day lag patterns of PM_2.5_ by applying the polynomial distributed lag model (DLM) with the same splines to control for the nonlinear time trend [34]. The reason that 15 days were chosen as a lag period was to assess the short-term effects of PM_2.5_.

All statistical analyses were performed using the packages mgcv and dlnm in the software R 3.6.1 (R Foundation for Statistical Computing, Vienna, Austria). Statistical graphing was achieved using the packages plotrix and ggplot2 in R. Two-sided statistical tests were performed, and the regression coefficients with a *P*-value < 0.05 were considered statistically significant. To check the robustness of our model, a sensitivity analysis was applied by changing the knots in the smoothness of time and adding the co-exposed pollutants NO_2_ and SO_2_ in the models.

### 2.4. Ethical Consideration and Data Availability

The study is an ecological and observational study, based on the data from population-based registers in Shanghai. No personal identification was disclosed in our data. The study was approved by the Ethical Review Committee of the SCDC (approval number: SCDC2016-08).

The use of the data was under the agreement between the Institute of Environmental Medicine, Karolinska Institutet, Sweden and the SCDC within a bilateral collaboration framework. The data were not publicly available but may be available upon reasonable request and with permission of the SCDC (xiatian@scdc.sh.cn).

## 3. Results

### 3.1. Characteristics of CVD Mortality, PM_2.5_ Concentration, and Meteorological Conditions

**Figure 1:**
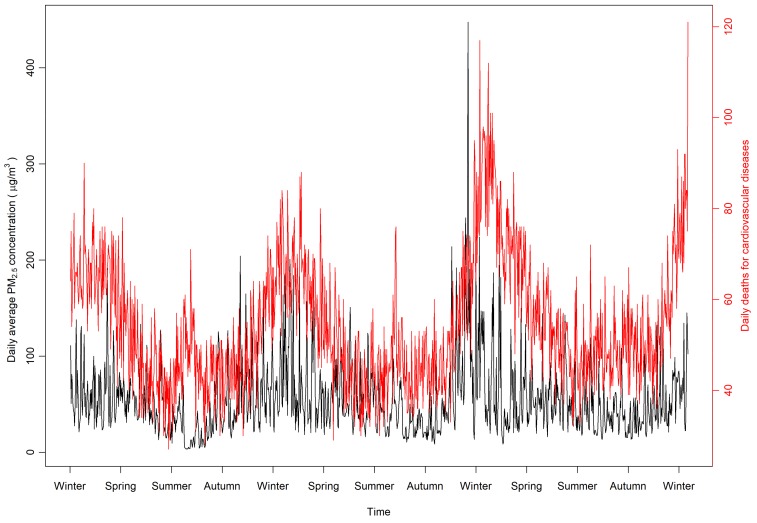
Time trends of daily ambient fine particulate matter (PM_2.5_) concentrations and cardiovascular disease (CVD) deaths.

During the study period, a total of 59,486 CVD deaths occurred in Shanghai, with a mean of 54.3 daily deaths and a median of 51. Distribution of daily CVD deaths approximated to a quasi-Poisson distribution with a mean of 54.3 and an overdispersion index of 1.47. Overall, the annual average concentrations of PM_2.5_ in Shanghai was 55.0 µg/m^3^, with a median value of 45.5 µg/m^3^. In general, the daily average PM_2.5_ concentrations and the numbers of daily deaths show similar seasonal trends. For both measurements, the values presented high in cold seasons and low in the warm seasons (Figure 1). The predicted daily death counts by the GAM, including PM_2.5_, DOW, SWT, and the interactions between PM_2.5_ and SWT, indicate that 71.2% of the deviance can be explained by the model.

The meteorological features of the six SWTs were described in detail in our previous study and are also provided in the Supplementary Table S1 [26]. In general, the annual average temperature in Shanghai is 17.2 °C, and the precipitation is about 1190 mm a year. The humid SWT, including warm humid, cold humid, and moderate humid days, occurs nearly half (49.8%) of the days, while dry weather occurs for the other half. Average levels of PM_2.5_, SO_2_, and NO_2_ by SWT are provided in the Supplementary Table S2.

### 3.2. Effects of PM_2.5_, SWT, and Their Interactions on CVD Mortality

The effects of PM_2.5_ and SWT on CVD deaths are shown in Table 1. Both the models with (adjusted R^2^ = 0.704) and without (adjusted R^2^ = 0.705) the co-exposed pollutants SO_2_ and NO_2_ show similar statistically significant associations between PM_2.5_ and CVD deaths. However, no statistically significant association was found between SO_2_ (risk ratio (RR) = 1.0007, 95% CI: 0.9993, 1.0021) or NO_2_ (RR = 0.9998, 95% CI: 0.9990, 1.0005) and CVD deaths in our data (Table 1). Without adjusting for SO_2_ and NO_2_, each 10 μg/m^3^ increase in PM_2.5_ concentration was associated with 1.26% (95% CI: 0.40%, 2.12%) increase in daily CVD mortality (RR = 1.0126, 95 CI: 1.0040, 1.0212; Table 1).

None of the SWTs alone had a statistically significant association with CVD deaths. Nevertheless, humid weather tended to have a higher-magnitude association with CVD deaths. Compared to hot dry SWT, cool humid SWT had positive association with mortality (RR = 1.037, 95% CI: 0.967, 1.112), followed by cold humid SWT (RR = 1.014, 95% CI: 0.944, 1.089). In contrast, cold dry SWT had a negative association (RR = 0.989, 95% CI: 0.919, 1.065).

In terms of interaction between the air pollutant and SWT, the interaction between PM_2.5_ and cold SWT had the largest association compared with other interactions. Statistically significant interactions were found between PM_2.5_ and cold dry SWT (RR = 0.985, 95% CI: 0.976, 0.995) and cold humid SWT (RR = 0.986, 95% CI: 0.976, 0.995) (Table 1).

### 3.3. Lag Effect of PM_2.5_ on CVD Mortality

**Figure 2:**
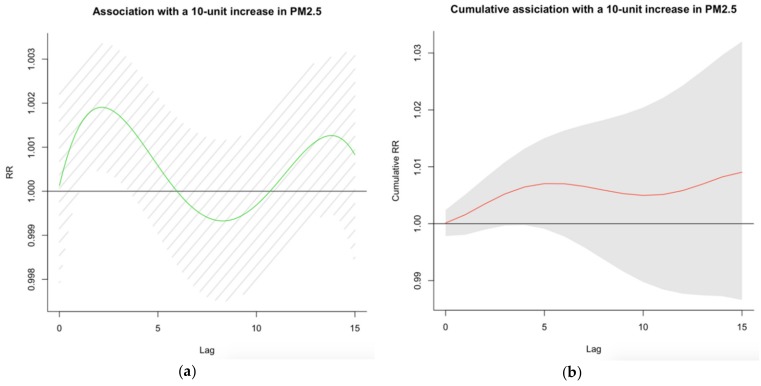
(**a**) Fifteen-day lag patterns and (**b**) cumulative lag patterns of risk for PM_2.5_-associated CVD mortality. The x-axes are lag days, ranging from lag day 0 to lag day 15; the y-axes are risk ratio (RR) or cumulative RR with 95% confidence interval for daily CVD mortality per 10 µg/m^3^ increase in PM_2.5_ concentration.

The lag effects of exposure to PM_2.5_ on CVD mortality based on the polynomial DLM analysis are shown in Figure 2a,b and Table 2. Figure 2a illustrates the single-day lag effects of PM_2.5_ on with CVD mortality associated with a 10 µg/m^3^ increase in PM_2.5_. Figure 2b illustrates the cumulative lag effects on CVD mortality associated with a 10 µg/m^3^ increase in PM_2.5_ concentration.

Generally, the associations of PM_2.5_ with CVD deaths presented a tri-phase pattern within the 15 days, as seen in Figure 2a. Statistically significant effects of PM_2.5_ in single lag day on CVD mortality were observed between lag day 1 and day 3, and the largest magnitude association on the second and the third lag days (RR = 1.0019, 95% CI: 1.0004–1.0033; RR = 1.0017, 95% CI: 1.0003–1.0032, respectively). A harvesting effect [35], i.e., the decrease in overall CVD mortality during the subsequent days, was found between lag days 6 and 11 (Figure 2a,b). However, the effect was not statistically significant.

For cumulative lag effects, although no statistically significant effects was found for all lag days, the largest effects were found for lag days 14 and 15 (RR = 1.0082, 95% CI: 0.9873, 1.0296; RR = 1.0091, 95% CI: 0.9866, 1.0320, respectively) followed by lags 5 and 6 (RR = 1.0070, 95% CI: 0.9991, 1.0150; RR = 1.0070, 95% CI: 0.9978, 1.0164, respectively) (Table 2).

### 3.4. Sensitivity Analysis

Multi-pollutants including PM_2.5_, SO_2_, and NO_2_ were included in both the GAM analyses (Table 1) and polynomial DLM analyses (data not shown) to compare with the single-pollutant model. The results remained almost unchanged.

## 4. Discussion

Our study focuses on the short-term associations of ambient air pollutant PM_2.5_, SWT, and their interaction with daily CVD mortality between 2012 and 2014 in Shanghai, China. The average daily concentration of PM_2.5_ in Shanghai was 55.0 μg/m^3^, which is much higher than the reference value in the World Health Organization guidelines (10 μg/m^3^ for the annual mean) [36]. In this study, we found that a 10 μg/m^3^ increase in PM_2.5_ was associated with an overall 1.26% (95% CI: 0.40%, 2.12%) increase in acute CVD mortality, which is of higher magnitude than the results from other studies (between 0.63% and 0.80%) [6,10,37,38]. A multicenter meta-analysis in East Asia including Shanghai suggested that each 10 μg/m^3^ increase in PM_2.5_ was related to a 0.96% (95% CI: 0.46%, 1.46%) increase in cardiovascular mortality [39], which is similar to our study. The lag analysis in our study also indicates that the associations of PM_2.5_ with CVDs mortality are mainly within the first six days (from lag 0 to lag 5).

We also tried to assess whether there is an association between the SWT and CVD deaths. However, no statistically significant association was found for any SWT in our data. The results are similar to those for Shenzhen, another populous city in China, i.e., no detectable association was found in extreme weather and cardiovascular events [40]. It is of note that we found interactions between cold SWT and PM_2.5_ which were inversely associated with the risk of CVD deaths, i.e., cold weather was found to potentially compensate the adverse effect of PM_2.5_ on cardiovascular health. Some other research is consistent with our results. Vanos et al. conducted an investigation in ten cities in Canada on the combined effect of weather and air pollution, and found that cool days were less harmful for cardiovascular-related mortality [41]. In European countries, the relationship between air pollutants and cardiovascular mortality has been found to be overall positive and of higher magnitude at high air temperatures [42]. In Asia, a study in Korea also found similar modification effects [43].

Although the relationship between cardiovascular mortality and weather condition has been widely investigated, few studies have categorized weather types and analyzed the interaction with air pollution. SWT comprises more than just humidity, temperature, and diurnal temperature; instead, it includes the already known and potentially unknown meteorological characteristics relevant to mortality. If we only include individual meteorological variables as covariates in the regression models, bias could be produced in the model because of the exclusion of other variables. The statistically non-significant associations between the SWT alone and CVD deaths in our study may be due to the insufficient data or short time period, and the categorization of SWT also deserves further investigation

According to our lag analysis, the lag association of PM_2.5_ with CVD mortality kept statistically significant for about 4 days, from lag day 0 to day 3, while cumulative lag effects were not statistically significantly related to increased CVD mortality. The results suggest that the effects of PM_2.5_ is acute in terms of CVD deaths. Other studies discovered similar findings. Results from a national study in the U.S. showed on lag day 0 and 1, PM_2.5_ had the largest effect on CVDs [44]. Another study in Beijing also discovered PM_2.5_′s lag effects within 0–3 lag days [45]. Higher levels of gaseous components, including NO_2_ and SO_2_, have long been associated with poorer cardiovascular health [46,47]. A systematic review of the studies in China indicated that a 10 μg/m^3^ increase in the concentrations of NO_2_ and SO_2_ was associated with a 1.12% (95% CI: 0.76%, 1.48%) and 0.75% (95% CI: 0.42%, 1.09%) increase in cardiovascular mortality, respectively [10]. No statistically significant associations of NO_2_ and SO_2_ observed in our study may be due to the different modeling strategy (no SWT and interaction were used in previous studies) or the relative shorter time period in our study, which should be investigated using long-term time-series data [48].

Based on our findings, air pollution, in the top five risk factors for health, has increased CVD mortality with modification from SWT, and might cause heavy burden on health and social development in view of the large population in Shanghai, China. Though the association is subtle, air pollution and extreme weather together have a great excessive impact when considering the entire population. Learning from the Chinese experience [49,50], governments are supposed to continue strengthening control on air pollution, in order to reduce mortality and related costs. For individuals, it is suggested that the population should reduce travel in hot weather, especially with high levels of air pollution, and they can employ physical or dietary interventions, which offer protection against short-term air pollution-induced adverse cardiovascular responses [51,52].

There are some strengths and limitations to our study. To some extent, the study provides new evidence about environmental conditions associated with health, for few other studies investigated the interaction between air pollutants and weather conditions. Therefore, this study might contribute to the pool of global knowledge regarding this topic. Second, SWTs rather than individual meteorological variables should be used to control for weather conditions, which examine the biological effect as the organism’s response to weather conditions as a whole, rather than to individual meteorological variables [53]. However, there are also limitation in our study. First, because of different socioeconomic condition and climates, the lessons and experiences learned from our study are hard to generalize to other cities and populations. Secondly, because of the ecological nature of this study, the study design restricted us in exploring the causal nature of relationships. However, since weather can influence the whole region, it is hard to measure those variables at individual level sufficiently in a large population. Thirdly, the lag analysis was only conducted for PM_2.5_, because the method only takes continuous variables while SWT is a categorical variable. Fourth, no interaction between lags time for PM_2.5_ and SWT was examined in the lag analysis, because when too many interaction terms (in total 75 interaction terms for 15 lag days and 6 SWTs even when no cross-basis matrix for the interactions was taken into account) were included, the model failed to estimate the parameters. Considering the acute antagonistic effect found between PM_2.5_ and cold SWT, and the largest cumulative effect found for lag day 15, the PM_2.5_′s lag effects and their interaction with SWT deserve further investigation using appropriate models.

We recognize that the pollution data from a single monitoring station could not accurately reflect the distribution of the PM_2.5_ concentrations in Shanghai. However, we interpreted the associations between CVD deaths and the risk factors using the relative risk (RR), i.e., mortality rate ratio. Theoretically, the coefficient of PM_2.5_ in the regression model (or its exponential format RR) only depends on the change in PM_2.5_ levels but not on the absolute PM_2.5_ levels, and the coefficient *β*_0_ (or baseline mortality rate) was not of the interest in our study. Therefore, the estimation of PM_2.5_′s effect is valid when the change in PM_2.5_ from the single station might reflect the change in PM_2.5_ of the whole of Shanghai city, i.e., when the single station’s PM_2.5_ level increased, Shanghai city’s PM_2.5_ level also increased and vice versa. We would also like to point out that Shanghai is the city with the highest life expectancy in China and one of the highest in the world, therefore, we must explain the results cautiously and cannot generalize them to other parts of China.

## 5. Conclusions

Exposure to PM_2.5_ was associated with increased short-term risk of CVD deaths with lag effects within four days in Shanghai, China, while cold weather was associated with lower risk. Since the ecological study design restricted us in identifying causal relationships, in-depth prospective studies with individual level data are warranted in the future.

## Figures and Tables

**Table 1:** Associations of PM_2.5_, synoptic weather types (SWTs), and their interactions with daily cardiovascular mortality.

Variable	Risk Ratio (RR) for Cardiovascular Mortality			
	Model without SO_2_, NO_2_		Model with SO_2_, NO_2_	
	RR (95% CI)	*P*-Value	RR (95% CI)	*P*-Value
**PM_2.5_ (per 10 μ g/m^3^)**	**1.0126 (1.0040, 1.0212)**	**0.004**	**1.0121 (1.0027, 1.0215)**	**0.011**
SO_2_			1.0007 (0.9993, 1.0021)	0.310
NO_2_			0.9998 (0.9990, 1.0005)	0.544
**Synoptic weather types**				
Hot dry (Ref)				
Warm humid	0.9945 (0.9359, 1.0567)	0.858	0.9971 (0.9378, 1.0603)	0.927
Cold dry	0.9894 (0.9191, 1.0651)	0.778	0.9861 (0.9149, 1.0628)	0.714
Cool dry	0.9954 (0.9308, 1.0645)	0.893	0.9978 (0.9327, 1.0674)	0.948
Cool humid	1.0369 (0.9666, 1.1123)	0.311	1.0378 (0.9675, 1.1133)	0.300
Cold humid	1.0138 (0.9438, 1.0891)	0.707	1.0157 (0.9451, 1.0914)	0.672
**Day of week**				
Sunday (Ref)				
Monday	1.0269 (0.9963, 1.0585)	0.085	1.0269 (0.9963, 1.0585)	0.086
Tuesday	1.0178 (0.9874, 1.0491)	0.254	1.0182 (0.9876, 1.0497)	0.246
**Wednesday**	**1.0350 (1.0043, 1.0667)**	**0.025**	**1.0352 (1.0041, 1.0672)**	**0.026**
Thursday	1.0139 (0.9835, 1.0453)	0.373	1.0143 (0.9837, 1.0458)	0.363
Friday	1.0058 (0.9756, 1.0369)	0.712	1.0065 (0.9761, 1.0378)	0.681
**Saturday**	**1.0315 (1.0008, 1.0632)**	**0.044**	**1.0320 (1.0012, 1.0637)**	**0.042**
**Interaction**				
PM_2.5_ × Hot dry (Ref)				
PM_2.5_ × Warm humid	0.9924 (0.9822, 1.0027)	0.149	0.9926 (0.9822, 1.0030)	0.162
**PM_2.5_ × Cold dry**	**0.9853 (0.9761, 0.9946)**	**0.002**	**0.9852 (0.9758, 0.9946)**	**0.002**
PM_2.5_ × Cool dry	0.9905 (0.9802, 1.0009)	0.074	0.9904 (0.9800, 1.0009)	0.072
PM_2.5_ × Cool humid	0.9889 (0.9762, 1.0019)	0.093	0.9893 (0.9764, 1.0022)	0.104
**PM_2.5_ × Cold humid**	**0.9855 (0.9764, 0.9948)**	**0.002**	**0.9853 (0.9760, 0.9946)**	**0.002**

Ref, reference group; CI, confidence interval, Bold items, variables with statistical significance (*P*-value < 0.05).

**Table 2:** Risk ratio (95% confidence interval) of lag effects for single days and cumulative lag effects per 10 µg/m^3^ increase in PM_2.5_ concentration.

Lag Day	Single Day Effect	Cumulative Effect
Lag 0	1.0001 (0.9978, 1.0024)	1.0001 (0.9978, 1.0024)
**Lag 1**	**1.0005 (1.0000, 1.0029)**	1.0016 (0.9980, 1.0051)
**Lag 2**	**1.0019 (1.0004, 1.0033)**	1.0035 (0.9990, 1.0080)
**Lag 3**	**1.0017 (1.0003, 1.0032)**	1.0052 (0.9997, 1.0108)
Lag 4	1.0012 (0.9997, 1.0027)	1.0065 (0.9998, 1.0132)
Lag 5	1.0006 (0.9990, 1.0021)	1.0070 (0.9991, 1.0150)
Lag 6	1.0000 (0.9983, 1.0016)	1.0070 (0.9978, 1.0164)
Lag 7	0.9995 (0.9978, 1.0013)	1.0066 (0.9959, 1.0174)
Lag 8	0.9993 (0.9975, 1.0012)	1.0059 (0.9937, 1.0182)
Lag 9	0.9994 (0.9978, 1.0012)	1.0053 (0.9916, 1.0192)
Lag 10	0.9997 (0.9979, 1.0015)	1.0050 (0.9898, 1.0204)
Lag 11	1.0002 (0.9983, 1.0020)	1.0051 (0.9884, 1.0221)
Lag 12	1.0007 (0.9989, 1.0025)	1.0058 (0.9877, 1.0243)
Lag 13	1.0011 (0.9993, 1.0029)	1.0070 (0.9874, 1.0268)
Lag 14	1.0013 (0.9995, 1.0031)	1.0082 (0.9873, 1.0296)
Lag 15	1.0008 (0.9983, 1.0033)	1.0091 (0.9866, 1.0320)

Bold items, variables with statistical significance (*P*-value <0.05).

## Supplementary Materials

The following are available online at https://www.mdpi.com/1660-4601/17/3/1111/s1, Table S1: Meteorological characteristics of the six synoptic weather types (SWTs) in Shanghai, 2012–2014, Table S2: Daily average levels of the three pollutants by SWT in Shanghai, 2012–2014.
