# Supplementary material for: Short-Term Associations of Fine Particulate Matter and Synoptic Weather Types with Cardiovascular Mortality: An Ecological Time-Series Study in Shanghai, China
Source: Int J Environ Res Public Health. 2020 Feb 10;17(3):1111. doi: 10.3390/ijerph17031111 (PMC7038017; doi:10.3390/ijerph17031111)
Supplement: Supplementary file 1 [file ijerph-17-01111-s001.pdf]

Table S1. Meteorological characteristics of the six synoptic weather types (SWT) in Shanghai, 2012-2014.

SWT	Number of days	Pressure (kPa)	Temperature (°C)	Humid (%)	Precipitation (mm)	Wind speed (m/s)	Sunshine (hour)
Hot dry	167	100.6 ± 0.4	28.4 ± 4.0	62.0 ± 10.2	1.25 ± 4.55	3.41 ± 0.91	8.79 ± 2.76
Warm humid	214	100.8 ± 0.4	23.8 ± 3.8	79.9 ± 6.9	4.11 ± 8.28	2.24 ± 0.63	2.25 ± 32.77
Cold dry	158	102.4 ± 0.4	8.0 ± 5.1	60.8 ± 13.2	0.98 ± 3.43	2.82 ± 0.94	5.45 ± 3.39
Cool dry	225	101.7 ± 0.3	18.5 ± 3.8	66.4 ± 10.8	0.32 ± 1.35	2.68 ± 0.68	6.67 ± 3.30
Cool humid	107	101.1 ± 0.6	19.1 ± 6.1	82.3 ± 8.3	17.28 ± 25.26	3.83 ± 1.17	8.99 ± 1.76
Cold humid	225	102.5 ± 0.4	6.7 ± 3.2	72.0 ± 9.6	1.81 ± 4.39	2.48 ± 0.82	3.32 ± 3.36

Table s2. Daily average levels of the three pollutants by SWT in Shanghai, 2012-2014.

SWT	$PM_{2.5}$		SO_2		NO_2	
	Mean (SD)	Median [IQR]	Mean (SD)	Median [IQR]	Mean (SD)	Median [IQR]
Hot dry	41.2 (29.3)	37.6 [17.5, 56.9]	16.8 (8.9)	15 [10, 20]	36.2 (21.3)	32 [20, 50]
Warm humid	49.5 (30.1)	40.6 [29.4, 58.2]	14.0 (6.1)	12 [10, 16]	42.5 (19.1)	40 [27, 55]
Cold dry	82.8 (50.6)	72.3 [53.2, 99.0]	36.7 (15.4)	35 [23, 50]	66.7 (28.6)	63 [42, 94]
Cool dry	49.0 (30.4)	41.4 [29.4, 59.0]	18.3 (8.9)	15 [12, 21]	48.2 (20.7)	45 [31, 63]
Cool humid	40.4 (25.1)	38.6 [21.2, 54.2]	13.3 (5.8)	12 [9, 16]	38.6 (17.8)	37 [25, 49]
Cold humid	63.5 (42.9)	50.2 [32.9, 85.3]	26.1 (13.3)	21 [16, 35]	54.5 (26.8)	47 [34, 69]
